# Effects of Different Regeneration Scenarios and Fertilizer Treatments on Soil Microbial Ecology in Reclaimed Opencast Mining Areas on the Loess Plateau, China

**DOI:** 10.1371/journal.pone.0063275

**Published:** 2013-05-02

**Authors:** Junjian Li, Yuanming Zheng, Junxia Yan, Hongjian Li, Xiang Wang, Jizheng He, Guangwei Ding

**Affiliations:** 1 Institute of Loess Plateau, Shanxi University, Taiyuan Shanxi, China; 2 State Key Laboratory of Urban and Regional Ecology, Research Centre for Eco-environmental Sciences, Chinese Academy of Sciences, Beijing, China; 3 Biology Institute of Shanxi, Taiyuan Shanxi, China; 4 Chemistry Department, Northern State University, Aberdeen, South Dakota, United States of America; Dowling College, United States of America

## Abstract

The soil microbial community in reclaimed mining areas is fundamental to vegetative establishment. However, how this community responds to different regeneration scenarios and fertilizer treatments is poorly understood. This research evaluated plant and soil microbial communities from different regeneration scenarios and different fertilizer treatments. Regeneration scenarios significantly influenced soil bacterial, archaeal, and fungal rDNA abundance. The ratios of fungi to bacteria or archaea were increased with fertilizer application. The diversity of both plants and microbes was lowest in *Lotus corniculatus* grasslands. Regeneration scenario, fertilizer treatment, and their interaction influenced soil microbial richness, diversity and evenness indices. Labile carbon pool 2 was a significant factor affected plant and microbe communities in July, suggesting that plants and microbes may be competing for nutrients. The higher ratios of positive to negative association were found in soil bacteria and total microbe than in archaea and fungi. Stronger clustering of microbial communities from the same regeneration scenario indicated that the vegetative composition of regeneration site may have a greater influence on soil microbial communities than fertilizer treatment.

## Introduction

The considerable growth of the mining industry in China over the last few decades has generated a vast amount of solid waste, which occupies a huge area of land [1]. In 2006, regulations were initiated to reclaim abandoned coal mining areas for agriculture and forestry in Shanxi Province [1], [2]. It would be useful to have indicators to assess the effectiveness of reclamation treatments. Traditionally, the criteria for judging the effectiveness of such treatments largely involve vegetation coverage and diversity, soil erosion and physicochemical characteristics [3]. In contrast, soil microbial ecology is not well understood, and is important for establishment vegetation, soil formation and transformation of nutrients, especially during the initial stages of reclamation [4], [5].

The majority on studies of the effects of mine reclamation on microorganisms have focused on specific fungal groups (particularly mycorrhizae) [6], [7], and microbial biomass and activity [5], [8]–[10]. Studies have demonstrated that mycorrhizae can promote plant growth [11], improve soil structure [12] and maintain plant biodiversity and ecosystem stability [13]. Microbial activity has been assessed by measuring enzyme activity or metabolic quotient [14], [15]. Soil microbial biomass and activity have been found to be lower in reclaimed mining areas compared to undisturbed sites [3], [14]; and many investigations support the hypothesis that soil microbial biomass and activity increase as reclamation progresses [3], [9], [10]. However, the increasing trend was found in only recently reclaimed areas [15]. In addition, Mummey et al. [4] found that the total bacterial and fungal biomasses showed opposite trends as reclamation progressed.

Recently, genetic profiling methods have produced more information on soil microbial ecology compared to the cultivation of isolated microbes [16], [17]. The genetic characteristics of the soil microbial community in forest, grassland, and farmland were reported in literature [18]–[21]. But, few reports focus on soil microbial communities in reclaimed mining areas [5], [15]. Bacterial RISA (ribosomal RNA intergenic spacer analysis) fingerprinting has been used to demonstrate the effects of raw parent material properties on the microbial indices of reclaimed mine spoils [5]. Both bacterial and fungal DGGE (denaturing gradient gel electrophoresis) profiles have clearly shown differences between reclaimed and natural sites, but could not discriminate between time since treatment or treatment types [15]. T-RFLP (terminal restriction fragment length polymorphism) analysis is a highly reproducible genetic profiling method that has also been proven to be very useful for describing differences and changes in soil microbial community structures [22]–[24].

Bacteria, archaea, and eukaryota form the three main domains of the phylogenic tree of life [25]. The diversity and ecological significance of archaea has received rather less attention compared to bacteria and fungi [26]. In this study, we investigated bacterial, archaeal and fungal communities in the initial development stage of post-mining rehabilitation in east central Lvliang Mountains, China. We investigated the effects of vegetation type and fertilizer treatment on microbial diversity and composition. We hypothesized that both the vegetation and application of fertilizer would produce significant effects on soil microbial community structure. Fertilizer was only applied once just prior to planting in our study, therefore, we further predict that vegetation type will have a larger impact on microbial properties than fertilizer treatment.

## Materials and Methods

### 2.1. Study Sites

Research was conducted at Antaibao Mine in Northwest Plateau Loess (37°09.4′E; 111°31.1′N). The climate is terrestrial temperate, and the area experiences monsoons. Annual average precipitation is 480 to 510 mm, with rainfall occurring mainly in the summer. The annual average air temperature is about 10.1°C, with the lowest average minimum temperature in January (−5.6°C) and the highest average maximum temperature in July (23.7°C). The frost-free season ranges in length from 180 to 200 days. Undisturbed soil in the area is classified as Cumulic Anthrosol (WBR).

Ecological reconstruction was initiated on the abandoned land in 2009, and there were four regeneration scenarios including *Lotus corniculatus* (CO), *Medicago sativa* (SA) grasslands, *Pinus tabulaeformis* plantation (TA), and *Salix matsudana* -*Sabina chinensis* mixed forest (MF). **The grass seed was sow in rows (row space, 20** **cm)**, and the seeding rates of *L. corniculatus* and *M. sativa* were 5 and 10 kg·hm^−2^, respectively. 3-year-old *P. tabulaeformis* and *S. chinensis*, and 5-year-old *S.matsudana* seedling were planted 2 m apart. There were four fertilizer treatments in total: CK = no fertilizer added, IN = inorganic fertilizer (750 kg/hm^2^; N: P_2_O_5_ = 18∶12) added, OR = organic fertilizer (45 m^3^/hm^2^; N: 1.7%, Organic matter: 24.1%) added, and IO = combination of inorganic and organic fertilizer (375 kg/hm^2^ inorganic fertilizer +22.5 m^3^/hm^2^ organic fertilizer) to soils.

We only investigated plant species in the field; we did not sample plant species. There were no endangered or protected species involed in this study. The location is Antaibao Mine Company that is state-owned enterprise. No specific permits were required for the described field studies.

### 2.2. Study Plots Survey and Soil Sampling

To study the characteristics of the various plant communities under different restoration types, quadrats were set up in the study areas. Quadrats of 20 m×20 m, and 1 m×1 m were established in forest and grassland communities, respectively. There were 3 replications for each of the 16 treatments, resulting in a total of 48 quadrates. Sampling occurred on two occasions, April and July 2011. The cover, height, diameter at breast height (DBH), individual number for each tree species, and the cover and height for herbs were recorded in each quadrat [1).

From each site, 6 soil cores (7.5 cm diameter×10 cm depth) were randomly collected in the middle of rows and mixed from the profile of each plot and bulked [5]. Subsamples for microbial analysis were stored at 4°C until DNA extraction (<2 weeks). Subsamples for other analyses were air dried. All soil samples were sieved to pass 2 mm.

### 2.3. Soil Chemical Analysis

Values (g cm^−3^) for soil bulk density (BD) were obtained using the gravimetric method. Soil pH in dH_2_O was measured in subsets of field-moist soils at a soil:solution ratio of 1∶5 (g:ml) after 0.5–1 h. Soil organic carbon (SOC) was determined using the dichromate oxidation method. Total nitrogen (TN) was analyzed by the Kjeldahl method [27]. The labile and recalcitrant carbon pools were quantified by the two-step acid hydrolysis procedure with HS_2_O_4_ [28], [29].

### 2.4. DNA Extraction

DNA was extracted from 0.5 g fresh soil samples using Ultra-clean TM soil DNA Isolation Kits (MoBio Laboratory, USA) following the manufacturer’s protocol. The extracted DNA was eluted with **5** ml of solution S5 (MoBio Laboratories, cat. no. 12800-100) and stored at −20°C. The DNA extracts were 10-fold diluted and used as template with a final content of 1–10 ng in each reaction mixture to amplify soil bacterial, archaeal, and fungal rRNA genes.

### 2.5. PCR Assay for Real-time Quantification and T-RFLP

Real-time PCR was performed on an iCycler iQ 5 thermocycler (Bio-Rad). The probe TM189F and *Premix Ex Taq*™ (Takara Biotechnology, Japan) were applied into bacterial 16S rRNA gene quantitative assay. The reaction mixture for quantifying archaeal and fungal rRNA gene included SYBR® *Premix Ex Taq*™ with Green I (Takara Biotechnology, Japan). Primers were labeled at the 5′ end with the reporter dye FAM (6-carboxy-fluorescein) for T-RFLP analysis. The detailed information on primer, probe, and PCR condition are listed in table 1.

**Table 1:** Primers, probes and PCR conditions used for real-time PCR and T-RFLP.

Target group	Primer and probe	Sequence (5′–3′)	Reaction system	Thermal profile	Reference
Real-time PCR					
Bacteria	Primer Bact1369FB	CGGTGAATACGTTCYCGG	25 µl:12.5 µl *Premix Ex Taq*™, 1 µl BSA, 0.5 µl each primer and probe, 2 µl template, 8 µl H_2_O	10 s at 95°C for initial denaturation;35 cycles of 15 s at 95°C, 1 min at 56°C	[55]
	Primer Prok1492R	GGWTACCTTGTTACGACTT			
	Probe TM1389F	CTTGTACACACCGCCCGTC			
Archaea	Primer Ar364aF	CGGGGYGCASCAGGCGCGAA	25 µl: 12.5 µl of SYBR® *Premix Ex Taq*™, 1 µl BSA, 0.5 µl each primer, 2 µl template, 8.5 µl H_2_O	30 s at 94°C for initial denaturation;40 cycles of 20 s at 94°C, 30 s at 59°C,and 30 s at 72°C	[56]
	Primer Ar934b	GTGCTCCCCCGCCAATTCCT			
Fungi	Primer NS1	GTAGTCATATGCTTGTCC	25 µl: 12.5 µl of SYBR® *Premix Ex Taq*™, 1 µl BSA, 0.5 µl each primer, 2 µl template, 8.5 µl H_2_O	3 min at 95°C for initial denaturation; 40 cycles of 10 s at 95°C, 30 s at 55°C, and 1 min at 72°C	[57]
	Primer FUNG	CATTCCCCGTTACCCGTTG			
PCR for T-RFLP					
Bacteria	Primer 27F-FAM	GAGTTTGATCCTGGCTCAG	50 µl: 5 µl 10×PCR buffer (MgCl_2_, 2 mM), 4 µl 2.5 mM dNTPs, 0.5 µl EX-Taq polymerase (5 U µl^−1^), 1 µl each primer, 1 µl BSA, 4 µl template, 33.5 µl H_2_O(Reaction system for bacteria, archaea and fungi)	5 min at 94°C; 35 cycles of 45 s at 94°C,45 s at 54°C, 72°C for 90 s; 10 min at 72°C.	[58]
	Primer 1492R	ACGGCTACCTTGTTACGACT			
Archaea	Primer Ar364aF	CGGGGYGCASCAGGCGCGAA		35 cycles of 45 s at 94°C, 45 s at 58°C, 60 sat 72°C; 10 min at 72°C.	[56]
	Primer Ar934b-FAM	GTGCTCCCCCGCCAATTCCT			
Fungi	Primer NS1-FAM	GTAGTCATATGCTTGTCC		5 min at 94°C; 35 cycles of 30 s at 94°C, 30 sat 56°C, 60 s at 72°C; 10 min at 72°C.	[57]
	Primer FUNG	CATTCCCCGTTACCCGTTG			

### 2.6. T-RFLP Analysis

All PCR products were verified using 1% agarose gel electrophoresis and purified with Wizard® SV Gel and PCR Clean-Up System (Promega, USA). Purified products were digested in separate reactions with restriction endonucleases *Hha*I, *Hae*III and *Msp*I (Takara Biotechnology, Japan) and incubated at 37°C for 3 h in the manufacturer’s recommended reaction buffer. Digestions were in a total volume of 25 µl, including 4 U of enzyme and about 500 ng of DNA. The digestion products were further purified, and a portion was mixed with deionized formamide and the internal standard GeneScan-ROX1000 (bacteria)/LIZ 500 (archaea and fungi) (Applied Biosystems). The mixtures were denatured for 3 min at 95°C, and the DNA fragments were size separated using a 3130xl Genetic Analyzer (Applied Biosystems).

### 2.7. Statistical Analysis

The effects of regeneration scenario and fertilizer treatment on plant and soil microbe characteristics were examined with separately one-way analyses of variance (ANOVA). A three-way ANOVA was applied to analyses the effects of season, regeneration scenario and fertilizer treatment on soil microbial communities. Plant species-pairs and microbial RF (Restricted fragment)-pairs association were analyzed by Spearman rank correlation. These statistical analyses were performed using SPSS 13.0 for Windows.

Plant species importance values (IV) for each quadrat were calculated using the following formulas [2]:
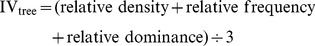
(1)

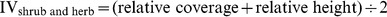
(2)

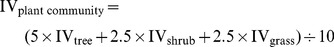
(3)


To reveal their variation among different restoration types, plant species, and RF richness, diversity and evenness in each quadrat were determined. Four species diversity indices were employed:

- Species number as the richness index (*S*)
- Shannon–Wiener diversity index:
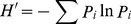
(4)
- Simpson diversity index:
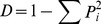
(5)
- Pielou evenness index:
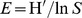
(6)

*S* is the number of plant species. *P_i_* is the relative importance value of plant species i. 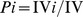; IV_i_ and IV are the importance value of species i and all plant species in a quadrate, respectively [2]. For soil microbial community, *S* is the number of RF and *P_i_* is the relative ratio of RF i [30].

A matrix of IVs for plant and matrices of RFs ratios for bacteria, archaea, fungi and total microbes were used as the basis of the community analysis. According to quantification PCR, the ratios of logarithm of the bacterial, archaeal and fungal rRNA gene copies approximate to 4∶3∶3. 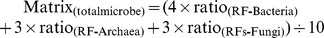. A matrix of environmental values was also established, which were used to analyze the relationships between samples and environmental factors using canonical correspondence analysis (CCA). These calculations were carried out using the CANOCO 4.5 [31], and the plant and microbial data were logarithmic transformed for the analysis. The significance level (P<0.05) between species and environmental data was used the Monte Carlo permutation test (Number of permutation is 499), and the significant environmental factors were applied into final CCA.

## Results

### 3.1 Soil Chemical Properties

Fertilizer treatments had significant effects (P<0.05) on soil pH in all vegetation types except SA grassland (Table 2). Soil pH in mixed forest was higher than in other regeneration scenarios under both CK and IN treatments. However, no significant differences (P>0.05) were found between scenarios treated with IO or OR fertilizer. Fertilizer treatments significantly (P<0.05) increased soil organic carbon in SA grassland and less significant effects of fertilizer treatments on soil organic carbon were demonstrated in other regeneration scenarios. In most cases, there were significantly (P<0.05) higher levels of soil organic carbon in CO and MF sites with the same fertilizer treatment. OR fertilizer significantly improved soil nitrogen content in the two grasslands and only inorganic fertilizer affected soil nitrogen in the mixed forests, but there were no significant differences among fertilizer treatments in the TA plantation. No significant differences in soil nitrogen were found between regeneration scenarios that did not have any fertilizer treatment. Under IN and IO fertilizer treatments, soil nitrogen from SA grassland was significantly (P<0.05) lower than that of the other vegetation types. Fertilizer did not produce significant effects (P<0.05) on labile carbon pools 1 and 2 (LC1 and LC2). Under the same fertilizer treatment, a significantly higher LC1 was found in CO grassland, and a significantly higher LC2 was found in MF. Different regeneration scenarios and fertilizer treatments both significantly (P<0.05) influenced soil recalcitrant carbon levels.

**Table 2:** Soil pH, bulk density, organic carbon, nitrogen, labile carbon pool 1 and 2 (LC1 and LC2), and recalcitrant pool carbon (RC) in the reclaimed mining area in July.

	pH	Bulk density	Organic carbon	Nitrogen	LC1	LC2	RC
CO-CK	7.96±0.01 bA	1.20±0.04aB	2.61±0.13aB	0.33±0.01aA	1.15±0.19aB	0.36±0.25aA	1.10±0.09abAB
CO-IN	7.87±0.03 aA	1.23±0.04aA	2.49±0.23aAB	0.37±0.02aB	1.47±0.24aB	0.42±0.16aA	0.60±0.17aA
CO-IO	7.92±0.04 abA	1.25±0.02aA	2.18±0.40aB	0.34±0.01aB	1.15±0.18aB	0.15±0.09aA	0.88±0.45aAB
CO-OR	7.89±0.02 aA	1.16±0.08aA	3.22±0.19bC	0.43±0.02bB	1.28±0.29aB	0.47±0.24aA	1.47±0.30bA
SA-CK	8.01±0.06 aA	1.01±0.05aA	2.29±0.28abB	0.35±0.01aA	0.58±0.19aA	0.44±0.09aA	1.28±0.31bB
SA-IN	7.93±0.04 aAB	1.14±0.11abA	2.14±1.06abAB	0.29±0.01aA	0.70±0.10aA	0.35±0.05aA	1.69±0.05cC
SA-IO	8.05±0.17 aA	1.32±0.09cA	1.29±0.03aA	0.25±0.01aA	0.45±0.06aA	0.31±0.10aAB	0.54±0.05aA
SA-OR	8.06±0.28 aA	1.26±0.05bcAB	2.61±0.23bAB	0.46±0.04bB	0.67±0.10aA	0.49±0.09aA	1.45±0.24bcA
TA-CK	7.95±0.01 bA	1.35±0.04aC	1.68±0.23aA	0.34±0.04aA	0.69±0.19aA	0.27±0.09aA	0.72±0.18aA
TA-IN	7.87±0.06 aA	1.27±0.02aA	1.68±0.13aA	0.36±0.03aB	0.50±0.19aA	0.54±0.16aA	0.64±0.20aA
TA-IO	7.88±0.01 aA	1.33±0.17aA	1.86±0.08abB	0.35±0.02aB	0.70±0.19aA	0.50±0.18aB	0.67±0.15aA
TA-OR	7.91±0.02 abA	1.34±0.06aB	2.21±0.27bA	0.36±0.04aA	0.77±0.11aA	0.48±0.09aA	0.95±0.39bA
MF-CK	8.24±0.11 bB	1.17±0.07aB	2.73±0.34aB	0.33±0.00aA	0.72±0.13aA	0.89±0.16aB	1.11±0.41aAB
MF-IN	7.97±0.03 aB	1.29±0.18aA	3.09±0.27abB	0.52±0.03bC	0.84±0.15aA	1.11±0.10aB	1.14±0.36aB
MF-IO	7.91±0.10 aA	1.26±0.06aA	3.56±0.15bC	0.36±0.02aB	1.07±0.11aB	1.15±0.23aC	1.34±0.23aB
MF-OR	8.03±0.04 aA	1.27±0.08aAB	3.10±0.42abB	0.31±0.02aA	0.94±0.11aA	0.95±0.10aB	1.21±0.44aA

Data are means ± standard deviations. Treatments with the same lower case letters are not significantly different from one another for the same regeneration scenario (P>0.05). Treatments with the same capital letters are not significantly different from one another for the same fertilizer treatment (P>0.05).

CO, SA, TA and MF respectively represent Lotus corniculatus, Medicago sativa, Pinus tabulaeformis and Salix matsudana–Sabina chinensis mixed forest.

CK, IN, IO and OR respectively represent no, inorganic, organic and a combination of inorganic and organic fertilizer added to soils.

### 3.2 Plant Community Composition and Diversity

**Figure 1:**
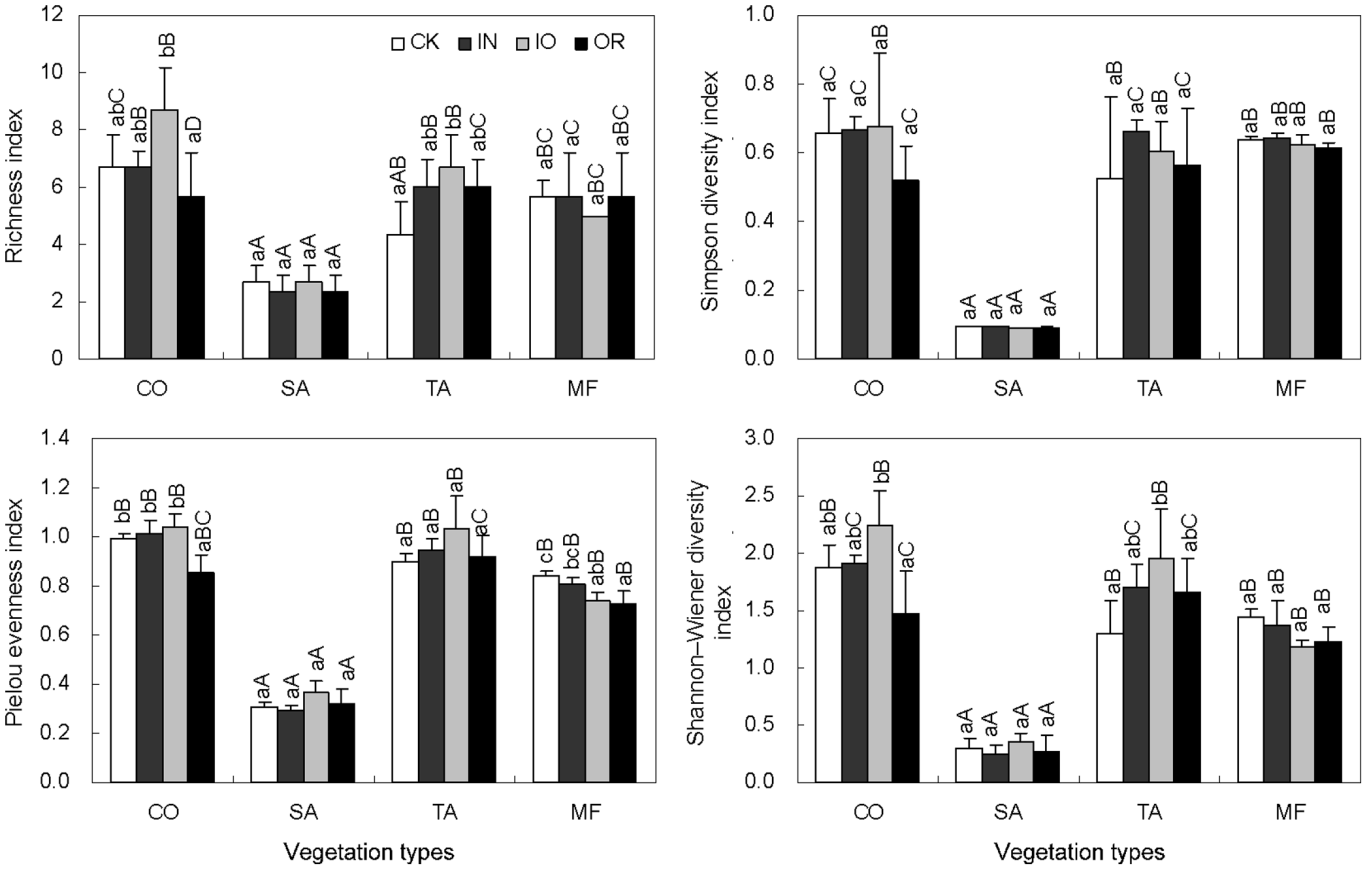
Plant species richness index, Shannon-Wiener diversity index, Simpson diversity index, and Pielou evenness index in the reclaimed mining area. Points show the means of three replicates, and vertical bars show standard deviations. Treatments with the same lower case letters are not significantly different from one another for the same regeneration scenario (P>0.05). Treatments with the same capital letters are not significantly different from one another for the same fertilizer treatment (P>0.05). CO, SA, TA, and MF represent *Lotus corniculatus, Medicago sativa, Pinus tabulaeformis,* and *Salix matsudana*–*Sabina chinensis* mixed forest. CK, IN, IO, and OR represent no fertilizer, inorganic, organic, and a combination of inorganic and organic fertilizer added to soils.

Plant community composition and the coverage of vegetation were investigated (Table S1). The vegetation coverage was lower in CO grassland than the other regeneration scenarios. IO fertilizer treatments significantly (P<0.05) improved the plant species richness and Shannon-Wiener diversity in CO and TA sites, but no significant effects were found in other regeneration scenarios (Figure 1). There were no significant (P>0.05) differences in the Simpson diversity index among fertilizer treatments. With the exception of SA grassland, fertilizer significantly (P<0.05) influenced the Pielou evenness index, although the effect was not consistent. Plant diversity indices from SA grassland were significantly lower than other sites treated with the same fertilizer.

### 3.3 Soil Bacterial, Archaeal, and Fungal Abundance

**Figure 2:**
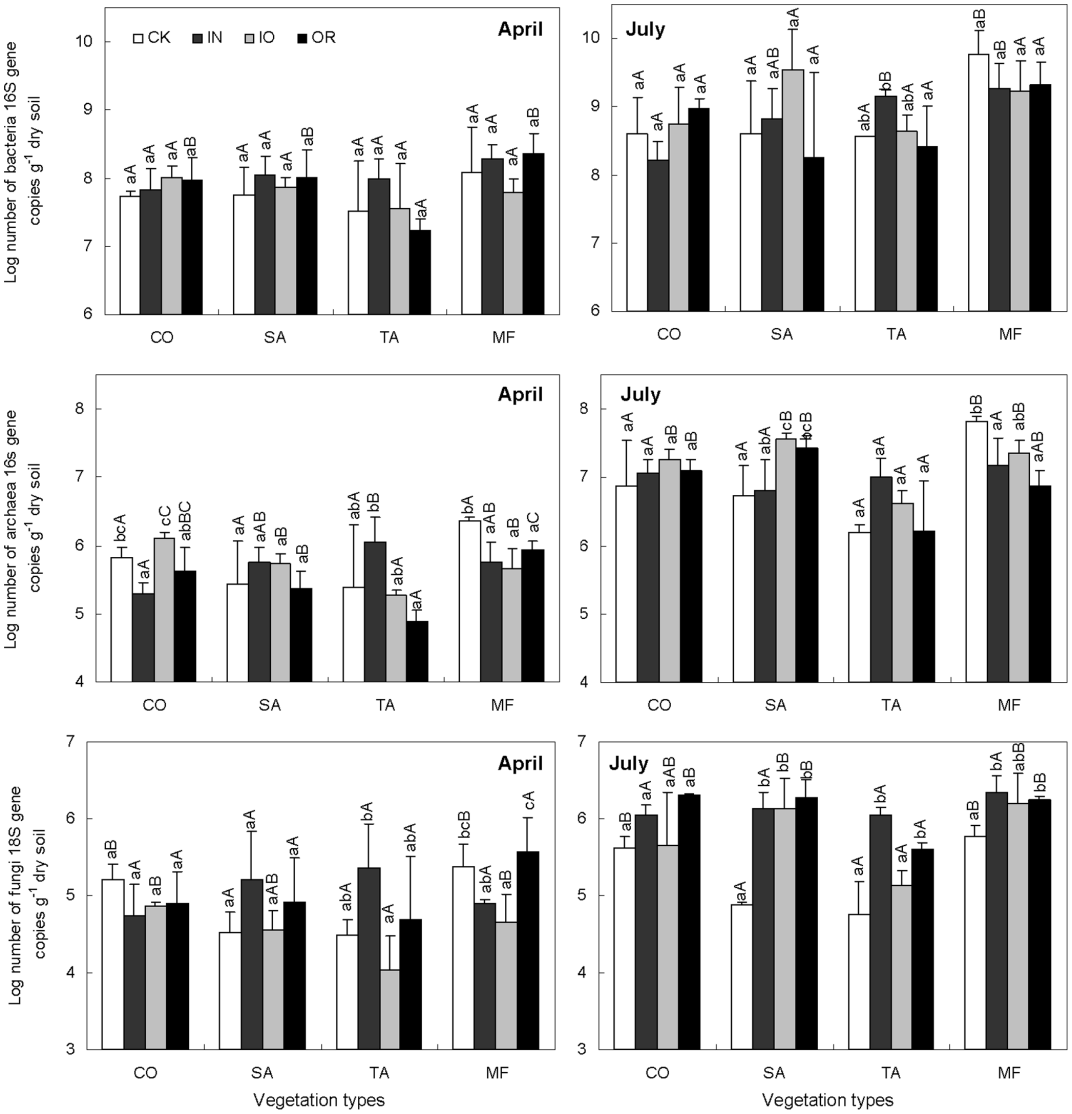
Abundance of soil bacteria, archaea, and fungi rRNA genes in the reclaimed mining area. Points show the means of three replicates, and vertical bars show standard deviations. Treatments with the same lower case letters are not significantly different from one another for the same regeneration scenario (P>0.05). Treatments with the same capital letters are not significantly different from one another for the same fertilizer treatment (P>0.05). CO, SA, TA, and MF represent *Lotus corniculatus, Medicago sativa, Pinus tabulaeformis,* and *Salix matsudana*–*Sabina chinensis* mixed forest. CK, IN, IO, and OR represent no fertilizer, inorganic, organic, and a combination of inorganic and organic fertilizer added to soils.

The seasonal effects on the abundance of soil microbial rRNA genes were significant (Figure 2). Fertilizer treatments produced no significant effects on soil bacterial 16S rRNA gene copy number, with the exception of the TA plantation in July. Either, there were no pronounced effects of fertilizer treatments on soil archaeal and fungal rRNA gene abundance in most cases. Different regeneration scenarios had a more obvious influence on the microbial rRNA gene abundance than fertilizer treatments, especially in archaea. In addition, the differences were more visible between regeneration scenarios in July than in April. In July, the log ratios of bacteria: fungi and archaea: fungi under SA and MF were lower with fertilizer treatments (Figure S1), which indicated that fertilizers show more positive effects on the growth of fungi than bacteria and archaea.

### 3.4 Soil Microbial T-RFLP Profiles and Diversity

T-RFLP profiles of soil bacterial, archaeal, and fungal rRNA genes from study sites were produced using the endonuclease enzymes *Hha*I, *Hae*III and *Msp*I. The effects of sampling date, regeneration scenarios, and fertilizer treatments on microbial diversity indices produced by *Hha*I are listed in Table 3.

**Table 3:** Results of three-way ANOVA showing the effects of season, regeneration scenarios and fertilizer treatments for soil bacteria, archaea, fungi and total microbes RFs richness index (*S*), Shannon-Wiener diversity index (*H’*), Simpson diversity index (*D*) and Pielou evenness index (*E*).

	Bacteria				Archaea				Fungi				Total microbes			
	*S*	*H’*	*D*	*E*	*S*	*H’*	*D*	*E*	*S*	*H’*	*D*	*E*	*S*	*H’*	*D*	*E*
Season	ns	ns	**	ns	**	**	**	**	**	*	ns	**	**	**	**	**
Scenarios	**	**	**	**	**	**	**	**	**	**	ns	ns	ns	ns	ns	ns
Fertilizer	ns	*	**	*	**	**	**	ns	*	ns	ns	ns	**	*	ns	ns
Season × Scenarios	ns	ns	ns	*	*	**	**	**	**	**	ns	*	*	ns	ns	ns
Season × Fertilizer	ns	ns	**	*	*	**	**	**	ns	ns	ns	*	ns	*	*	**
Scenarios × Fertilizer	ns	ns	*	ns	ns	*	*	**	*	*	**	*	*	ns	ns	ns
Season × Scenarios × Fertilizer	ns	ns	ns	ns	ns	**	**	**	**	ns	*	*	*	ns	ns	ns

** Effect is significant at the 0.01 level;

* Effect is significant at the 0.05 level. ns, effect is not significant.

When soil bacteria 16S rRNA genes were restricted using *Hha*I, the 77 bp RF had the highest relative abundance and there were RFs unique to each regeneration scenario. In T-RFLP profiles of soil archaeal 16S rRNA restricted using *Hha*I, the 320 bp RF had the highest relative abundance at all sites. Fragments of 136 and 141 bp were found in regeneration areas where trees grew, but not in grasslands; and the fragments lower than 88 bp were only observed in fertilizer treatments. Both regeneration scenario and fertilizer treatment demonstrated an influence on T-RFLP profiles of soil fungi 18S rRNA restricted using *Hha*I enzyme (Figure S2).

Soil bacterial and total microbial diversity indices inferred from fragments restricted using *Hha*I and *Msp*I were more liable to be influenced by sampling date, regeneration scenarios, fertilizer treatments and their interaction, than those restricted using *Hae*III(Table 3 and Table S2). Soil archaeal diversity indices from RFs produced using *Hha*I appeared to be more sensitive to these factors. Most of the diversity indices for fungal fragments restricted using *Msp*I were not significantly influenced by vegetation type or fertilizer treatment. Sampling date was the most significant factor affecting microbial diversity, and followed by regeneration scenario, then fertilizer treatment. The Pielou evenness index (*E*) and richness index (*S*) were the most and least susceptible for these factors, respectively.

### 3.5 Spearman Rank Correlation Test for Inter-species Correlation

Plant species-pair and microbial inter-RF ratios of positive and negative association of the Spearman rank correlation test are listed in Table S3. Plant species-pair ratios at the CO and TA sites were higher than in the other two treatments. Fertilizer treatments improved both plant and microbe ratios. Soil microbe inter-RFs ratios differed with regeneration scenarios. Inter-RF ratios of total soil microbes were the highest, and decreased in order from bacteria to fungi to archaea.

### 3.6 Effect of Environmental Factors on Plant and Soil Microbial Community Composition and Species Distribution

**Figure 3:**
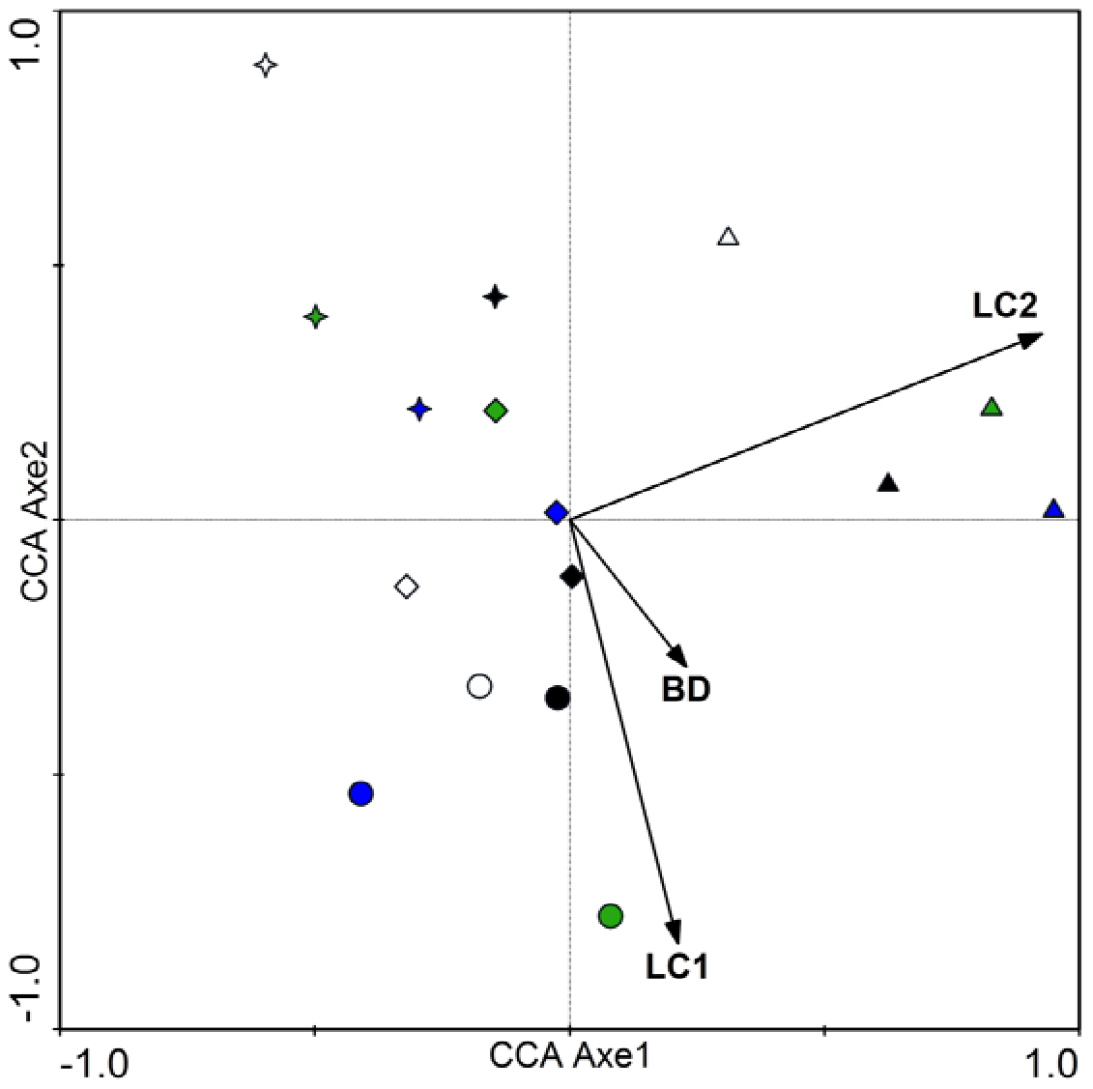
CCA ordination biplot of 16 quadrats and environmental factors for plant communities in the reclaimed mining area. Arrows indicate the direction and magnitude of measurable variables associated with plant community structures. Circle, Star, Diamond and Up-triangle symbol types respectively represent *Lotus corniculatus, Medicago sativa, Pinus tabulaeformis* and *Salix matsudana*–*Sabina chinensis* mixed forest. White, green, blue and black respectively represent no, inorganic, organic and a combination of inorganic and organic fertilizer added to soils. BD, LC1, and LC2 are bulk density, soil labile pool carbon 1 and 2, respectively.

The results of CCA showed that the eigenvalues of axes 1 and 2 was respectively 62.3% and 44.0%, and the first two axes explained 64.7% of species–environment relations (i.e. Plant-environmental data). Liable carbon pool and bulk density were significantly linked to the plant community variability (P<0.05), and positively correlated with the first axes (Figure 3).

**Figure 4:**
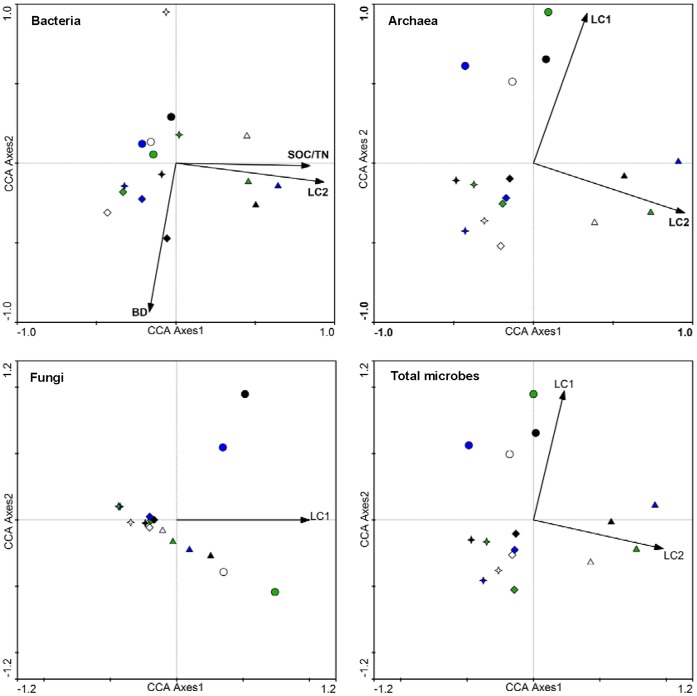
CCA ordination biplot of 16 quadrats and environmental factors for soil bacterial, archaeal, fungal, and total microbial communities in the reclaimed mining area in July. Arrows indicate the direction and magnitude of measurable variables associated with soil microbial communities structures. Circle, Star, Diamond and Up-triangle symbol types respectively represent *Lotus corniculatus, Medicago sativa, Pinus tabulaeformis* and *Salix matsudana*–*Sabina chinensis* mixed forest. White, green, blue and black respectively represent no, inorganic, organic and a combination of inorganic and organic fertilizer added to soils. BD, SOC/TN, LC1, and LC2 are the soil bulk density, ratio of soil organic carbon to total nitrogen, labile carbon pool 1 and 2, respectively.

The relationships between environmental factors and soil microbial communities, according to T-RFLP profiles restricted using *Hha*I, were shown by the CCA method (Figure S3, Figure 4). In April, the significant environmental factors that affected bacterial, archaeal, fungal, and total microbial communities were pH, ratio of soil organic carbon to nitrogen, bulk density and LC2 (Figure S3). Soil pH was positively correlated with the first axes in bacterial and total microbial communities; however, negative effects were shown in fugal communities. Bulk density was significant environmental factors for archaeal and fungal communities, and positively correlated with the second axes. In July, LC1 was positively correlated with the first two axes, but the contrary correlations were demonstrated between LC2 and the first two axes (Figure 4). Treatments from the same regeneration scenario tended to cluster together more than those from the same fertilization treatment. Similar results were found when CCA was applied to test relationships between environmental factors and microbial RFs restricted using *Hae*III and *Msp*I enzymes (results not shown).

## Discussion

The growth of *L. corniculatus* demands high fertility soil, and we observed that fertilizer significantly improved plant coverage in CO grasslands. *M. sativa* has been widely planted in reclamation sites in the Loess Plateau due to its strong resistance to drought and barren soil [32]. In our study, *M. sativa* grew well, the coverage being over 90% with the available space almost used; other species, therefore, could not easily invade and plant richness, diversity and evenness was significantly lower in these grasslands (Figure 1). Possibly, due to the lower coverage in CO grasslands, plant diversity was higher as invasive species was able to establish easily. Inter-plant spacing provided gaps for shrub growth so plant diversity was also higher in TA and MF plantations.

A higher growth rate of vegetation results in more soil nutrients being consumed. Meanwhile, litter and dead roots are not degraded during the initial reclamation period. We observed higher coverage and above-ground biomass in SA than CO grasslands, and levels of soil organic carbon, total nitrogen, and labile carbon pool were lower in SA than CO grasslands (Table 2). Declining trends have been recorded in soil organic carbon, nitrogen, and microbial biomass in fast-growing poplar and *Eucalyptus* plantations [33]–[35].

In July, the different regeneration scenarios in our study demonstrated more obvious influences on soil microbial rDNA copy numbers (Figure 2). In addition, soil microbial RF richness and diversity were lowest in the SA grasslands where the richness and diversity of above-ground vegetation were also the lowest compared to other regeneration scenarios (Figure1). These results imply that these microbial communities are closely related to plant colonization. Previous studies have also supported the hypothesis that vegetation produces significant effects on the structuring of microbial communities during the pioneer stage of ecosystem development [36], [37]. The relationship between microbial reproduction and plant colonization involves competition for nutrients primarily during short-term rehabilitation programs [38]. However, nutritive symbiosis was chiefly for long-term rehabilitation potentially between regeneration scenarios and soil microbial communities via the chemical composition, quantities and botanical forms of different plant residues [39], [40].

In this study, fertilizer treatments were beneficial for vegetation establishment, particularly in the CO grasslands. Organic fertilizer produced significant (P<0.05) increases in soil organic and recalcitrant carbon levels and nitrogen content (Table 2). Fertilizers provide different substrates for soil microbial communities [41]–[43]. There were no significant differences between the three domains of soil microbial rDNA copy numbers (Figure 2). These results differ from previous studies where significant increases in soil microbial biomass due to fertilizer have been reported during the reclamation of barren soils or agricultural fields [44]–[46]. The difference may be explained by the fact that only a single application of fertilizer was in our study, whereas long-term application of fertilizers was used in other studies.

Soil pH was significantly (P<0.05) decreased as a result of fertilizer treatments, especially in the mixed forest (Table 2). However, the application rate of fertilizer used here did not produce significant detrimental effects on plant or microbial growth. Significant plant growth in part may have contributed to a decrease in pH via litter inputs, organic exudation, and proton extrusion [29], [47]. The much lower pH from sites treated with fertilizer could also be explained by the influence of fertilizer on microbial community structure, e.g., lower pH levels may improve the competitive advantage of soil fungi that are more suited to weak acid conditions compared to bacteria and archaea. Lower ratios of bacteria and archaea to fungi were found, which was consistent with other studies [48]. In addition, soil pH influenced soil bacterial and fungal composition across samples [37], [49] and was a significant environmental factor in determining soil bacterial and total microbial community composition in April (Figure S3).

Using a Monte Carlo permutation test in CCA, we showed that LC2 was a significant environmental factor affecting both plant and microbial communities in July. However, labile carbon was not the only significant environmental factor that affected soil microbial communities in April (Figure S3). The test also supported the view that there may be competition for nutrition between plants and microbes to meet their growth demands in infertile soil. In reclaimed coal mine soils, the development of microbial communities was shown to be stimulated by the presence of an easily available carbon source [9]. Previous studies have also shown that soluble carbon is a dominant influence on microbial communities in other conditions [50]–[52].

In our study, soil microbial diversity was more affected by the vegetative composition of the regeneration sites than by fertilizer treatments (Table 3). We also found microbial communities stronger clustering from similar regeneration scenarios than form similar fertilizer treatments (Figure 4, S3). These results suggest that regeneration scenarios have greater impacts on the microbial communities than fertilizer treatments. This is probably related to root exudates. In reclaimed post-mining sites near Sokolov (Czech Republic) and near Cottbus, Šourková *et al*. [14] reported that vegetation type played a more important role in the soil microbial community than substrate, which was mainly dependent on litter quality. We hypothesized that the degradation of litter would not be the primary substrate for soil microbial communities in 3-year-old reclaimed sites.

Higher ratios of positive to negative association from the Spearman rank correlation test indicate that biological community is more steady [53]. The low ratios listed in table S3 suggested that plant and soil microbe were still in the initial succession period. The higher ratios of bacteria suggested that bacteria would promote the succession of plant, archaea, and fungi, and play an important role in maintaining ecological stability. Our results are similar to Susyan *et al*. [54]. They reported that soil fungi clearly dominated the microbial communities later in the succession on abandoned arable soils, which suggested that the soil bacterial community prevailed during the initial period.

### Conclusions

The results partly confirmed our hypothesis that soil microbial communities were significantly influenced by regeneration scenarios, however, fertilizer treatments produced less significant influence on soil microbial communities. Regeneration scenarios produced a significant effect on soil microbial rDNA copy numbers and microbial communities. Season showed pronounced effects on soil microbial growth and composition. There may be nutrient competition between vegetation and microbes for their growth during initial rehabilitation period, but mutual benefits would be demonstrated between vegetation and soil microbe with succession progressed in reclaimed mining areas of Shanxi, China.

## Supporting Information

Figure S1 **Ratios of soil bacterial, archaeal and fungal log numbers of rRNA gene copy number in the reclaimed mining area.** Points show the means of three replicates, and vertical bars show standard deviations. Lower case letters indicate that the means are not significantly different among fertilizer treatments for the same regeneration scenario (*P*<0.05). Capital letters indicate that the means are not significantly different among regeneration scenarios for the same fertilizer treatment (*P*<0.05). CO, SA, TA and MF respectively represent *Lotus corniculatus, Medicago sativa, Pinus tabulaeformis* and *Salix matsudana*–*Sabina chinensis* mixed forest. CK, IN, IO and OR respectively represent no, inorganic, organic and a combination of inorganic and organic fertilizer added to soils.
(TIF)

Figure S2 **Relative fluorescence of soil bacteria, archaea and fungi populations measured by T-RFLP electropherogram target on rRNA gene sequences digested using** ***Hha*** **I restriction enzymes in the reclaimed mining area.** CO, SA, TA and MF respectively represent *Lotus corniculatus, Medicago sativa, Pinus tabulaeformis* and *Salix matsudana*–*Sabina chinensis* mixed forest. CK, IN, IO and OR respectively represent no, inorganic, organic and a combination of inorganic and organic fertilizer added to soils.
(TIF)

Figure S3 **CCA ordination biplot of 16 quadrats and environmental factors for soil bacterial, archaeal, fungal, and total microbial communities in the reclaimed mining area in April.** Arrows indicate the direction and magnitude of measurable variables associated with soil microbial communities structures. Circle, Star, Diamond and Up-triangle symbol types respectively represent *Lotus corniculatus, Medicago sativa, Pinus tabulaeformis* and *Salix matsudana*–*Sabina chinensis* mixed forest. White, green, blue and black respectively represent no, inorganic, organic and a combination of inorganic and organic fertilizer added to soils. BD, SOC/TN, and LC2 are the soil bulk density, ratio of soil organic carbon to total nitrogen, and labile carbon pool 2, respectively.
(TIF)

Table S1 **Plant community composition and coverage in reclaimed mining area.** CO, SA, TA and MF are respectively *Lotus corniculatus, Medicago sativa, Pinus tabulaeformis* and *Salix matsudana* -*Sabina chinensis* mixed forest. CK, IN, IO and OR are respectively no, inorganic, organic and combination of inorganic and organic fertilizer added to soils
(DOC)

Table S2 **Results of three-way ANOVA showing the effects of season, regeneration scenarios and fertilizer treatments for Soil bacteria, archaea, fungi and total microbe RFs richness index (** ***S*** **), Shanon-Wiener diversity index (** ***H’*** **), Simpson diversity index (** ***D*** **) and Pielou evenness index (** ***E*** **).** ** Effect is significant at the 0.01 level; * Effect is significant at the 0.05 level. ns Effect is not significant.
(DOC)

Table S3 **Ratios of positive and negative association from Spearman rank correlation test of the inter-species correlation of plant and inter-RFs correlation of soil bacteria, archaea, fungi and total microbes in the reclaimed mining area.** CO, SA, TA and MF represent *Lotus corniculatus, Medicago sativa, Pinus tabulaeformis* and *Salix matsudana*–*Sabina chinensis* mixed forest. CK, IN, IO and OR represent no, inorganic, organic and a combination of inorganic and organic fertilizer added to soils.
(DOC)
