# Correction: Effects of Different Regeneration Scenarios and Fertilizer Treatments on Soil Microbial Ecology in Reclaimed Opencast Mining Areas on the Loess Plateau, China

**DOI:** 10.1371/annotation/35e79a01-ad03-4e96-85e6-5def25d96581

**Published:** 2013-10-21

**Authors:** Junjian Li, Yuanming Zheng, Junxia Yan, Hongjian Li, Xiang Wang, Jizheng He, Guangwei Ding

Table 2 is missing the corresponding units of each column heading. Please see the corrected Table 2 here: 

**Figure:**
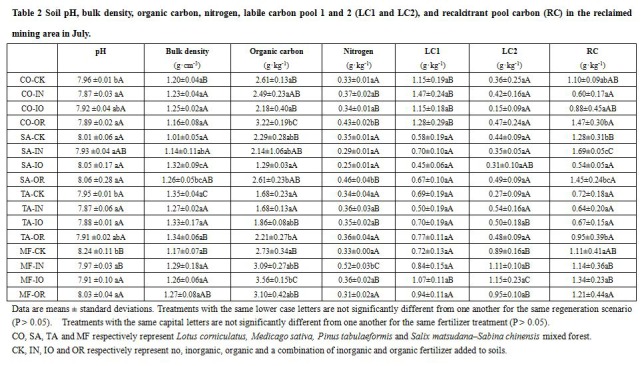


There was missing information from the funding section. The correct funding information reads: This work was financially supported by the Natural Science Foundation of China (41230857, 41271530 and 41025004), and the CAS/SAFEA International Partnership Program for Creative Research Teams of "Ecosystem Processes and Services", and the National Science and Technology Program (2012BAC10B04 and 2008BAD95B04). The funders had no role in study design, data collection and analysis, decision to publish, or preparation of the manuscript.

