# Supplementary figures and images for: Effects of Different Regeneration Scenarios and Fertilizer Treatments on Soil Microbial Ecology in Reclaimed Opencast Mining Areas on the Loess Plateau, China
Source: PLoS One. 2013 May 2;8(5):e63275. doi: 10.1371/journal.pone.0063275 (PMC3642173; doi:10.1371/journal.pone.0063275)

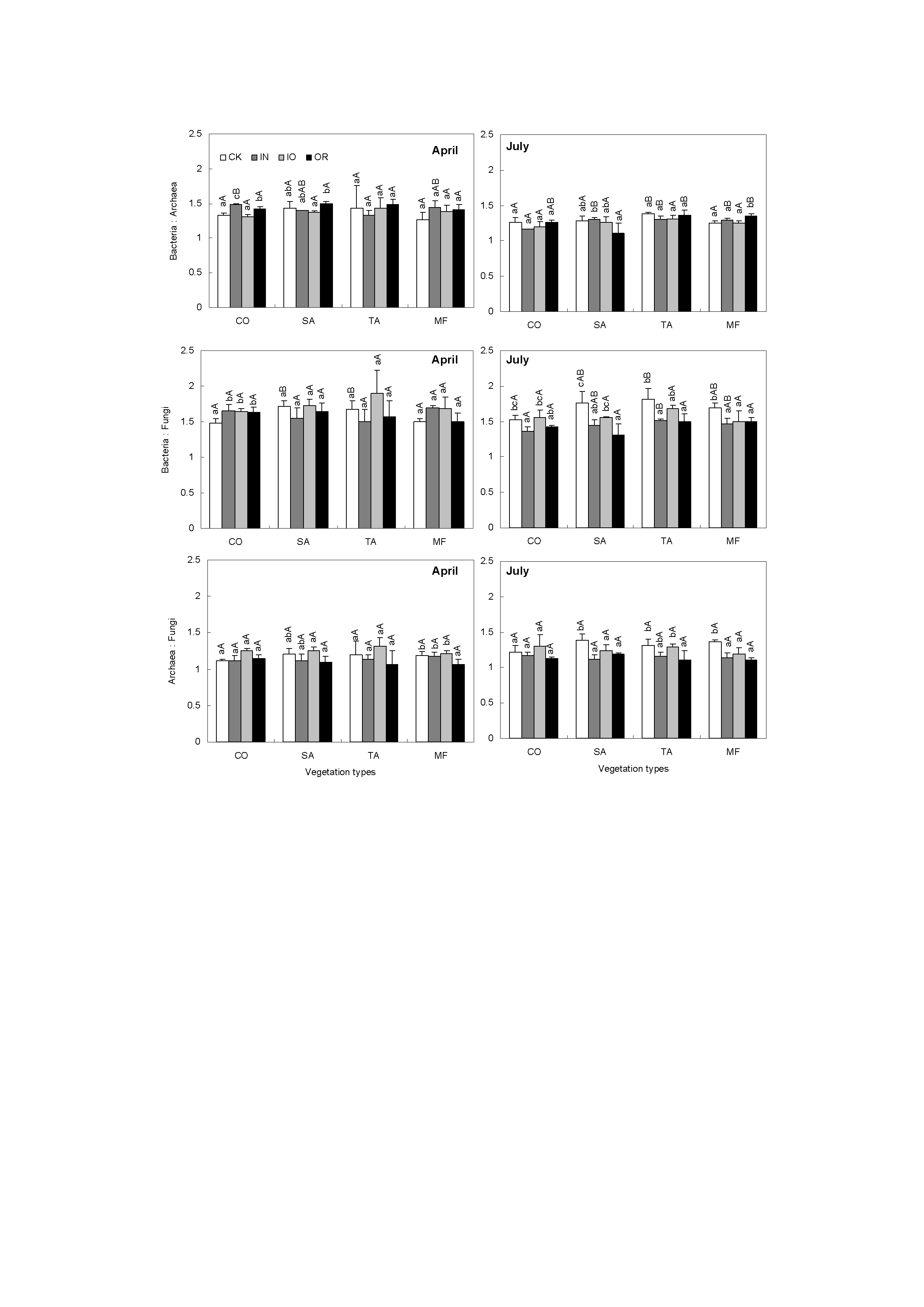

Supplement: Figure S1 — Ratios of soil bacterial, archaeal and fungal log numbers of rRNA gene copy number in the reclaimed mining area. Points show the means of three replicates, and vertical bars show standard deviations. Lower case letters indicate that the means are not significantly different among fertilizer treatments for the same regeneration scenario (P<0.05). Capital letters indicate that the means are not significantly different among regeneration scenarios for the same fertilizer treatment (P<0.05). CO, SA, TA and MF respectively represent Lotus corniculatus, Medicago sativa, Pinus tabulaeformis and Salix matsudana–Sabina chinensis mixed forest. CK, IN, IO and OR respectively represent no, inorganic, organic and a combination of inorganic and organic fertilizer added to soils. (TIF) [file pone.0063275.s001.tif]

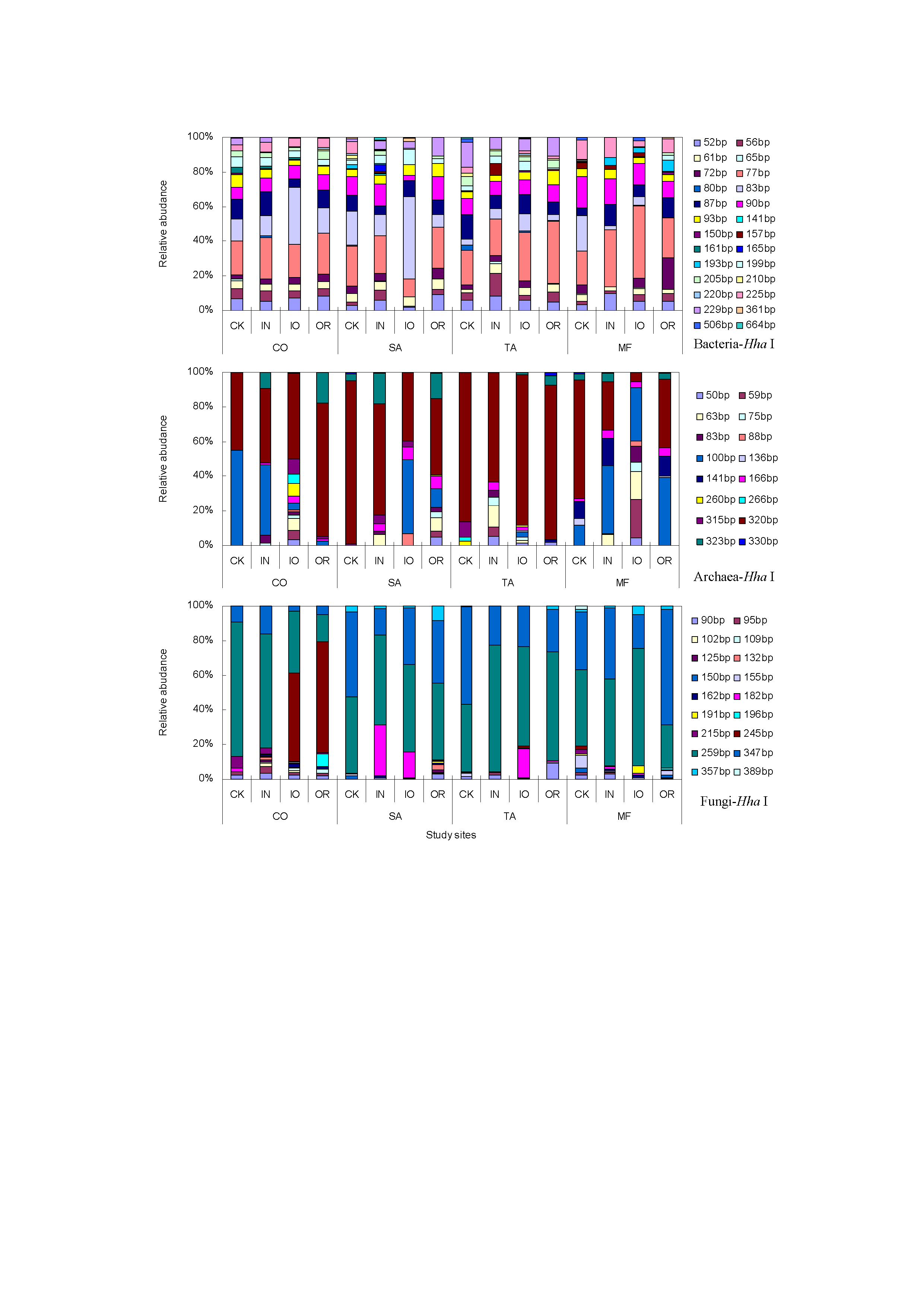

Supplement: Figure S2 — Relative fluorescence of soil bacteria, archaea and fungi populations measured by T-RFLP electropherogram target on rRNA gene sequences digested using Hha I restriction enzymes in the reclaimed mining area. CO, SA, TA and MF respectively represent Lotus corniculatus, Medicago sativa, Pinus tabulaeformis and Salix matsudana–Sabina chinensis mixed forest. CK, IN, IO and OR respectively represent no, inorganic, organic and a combination of inorganic and organic fertilizer added to soils. (TIF) [file pone.0063275.s002.tif]

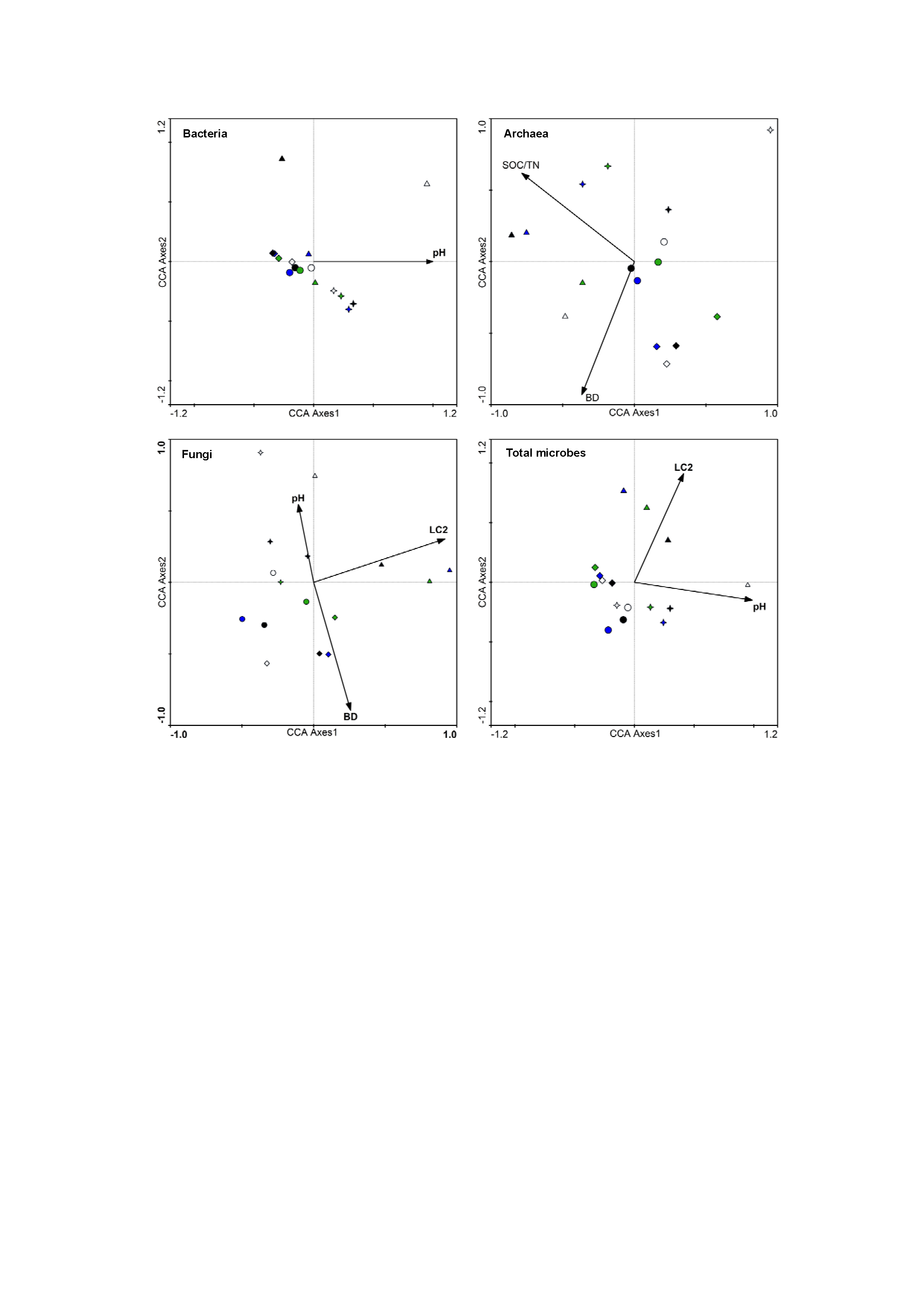

Supplement: Figure S3 — CCA ordination biplot of 16 quadrats and environmental factors for soil bacterial, archaeal, fungal, and total microbial communities in the reclaimed mining area in April. Arrows indicate the direction and magnitude of measurable variables associated with soil microbial communities structures. Circle, Star, Diamond and Up-triangle symbol types respectively represent Lotus corniculatus, Medicago sativa, Pinus tabulaeformis and Salix matsudana–Sabina chinensis mixed forest. White, green, blue and black respectively represent no, inorganic, organic and a combination of inorganic and organic fertilizer added to soils. BD, SOC/TN, and LC2 are the soil bulk density, ratio of soil organic carbon to total nitrogen, and labile carbon pool 2, respectively. (TIF) [file pone.0063275.s003.tif]
