# Supplementary material for: Effects of Different Regeneration Scenarios and Fertilizer Treatments on Soil Microbial Ecology in Reclaimed Opencast Mining Areas on the Loess Plateau, China
Source: PLoS One. 2013 May 2;8(5):e63275. doi: 10.1371/journal.pone.0063275 (PMC3642173; doi:10.1371/journal.pone.0063275)
Supplement: Table S1 — Plant community composition and coverage in reclaimed mining area. CO, SA, TA and MF are respectively Lotus corniculatus, Medicago sativa, Pinus tabulaeformis and Salix matsudana -Sabina chinensis mixed forest. CK, IN, IO and OR are respectively no, inorganic, organic and combination of inorganic and organic fertilizer added to soils (DOC) [file pone.0063275.s004.doc]

|  | **Plant community composition** | **Coverage** |
| --- | --- | --- |
| CO-CK | *L. cornioulatus, Ixeridium sonchifolium,* *Arabis pendula,* *Cynanchum chinense, Setaria viridis, Calamagrostiis pseudophragmites,* *Vicia amoena, Convolvulus arvensis, Artemisia sacrorum, Lagopsis supine, Conyza canadensis, Ixeridium chinense,* *Salsola collina* | 43% |
| CO-IN | *L. cornioulatus, A. pendula, C. pseudophragmites, Phragmites communis, A. sacrorum, I. chinense, Avena fatua*, *S. collina* | 77% |
| CO-IO | *L. cornioulatus, I. sonchifolium, A. pendula, Rehmannia glutinosa, C. chinense, Heteropappus hispidus, S. viridis, C. pseudophragmites, V. amoena, C. arvensis, A. sacrorum, L. supine, C. canadensis, I. chinense, S. collina* | 63% |
| CO-OR | *L. cornioulatus, A. pendula, R. glutinosa, H. Hispidus, C. Pseudophragmites, P. communis, C. arvensis, A. Sacrorum, I. Chinense, S. collina* | 82% |
| SA-CK | *M. sativa, I. sonchifolium, S. japonica, Medicago falcata, L. supine, Artemisia capillaries* | 95% |
| SA-IN | *M. sativa, I. sonchifolium, C. pseudophragmites, A. capillaries* | 95% |
| SA-IO | *M. sativa, M. falcate, Melilotus officinalis, C. pseudophragmites, A. capillaries* | 95% |
| SA-OR | *M. sativa, M. officinalis, C. pseudophragmites* | 95% |
| TA-CK | *P. tabulaeformis, I. sonchifolium, C. chinense, Lespedeza bicolor, Sonchus brachyotus, A. fatua, S. collina, Viola philippica* | 83% |
| TA-IN | *P. tabulaeformis, A. pendula, Artemisia hedinii, Cirsium japonicum , C. chinense, S. viridis, C. pseudophragmites, S. brachyotus, C. arvensis, C. canadensis, Cirsium setosum, I. chinense, S. collina* | 86% |
| TA-IO | *P. tabulaeformis, A. pendula, A. hedinii, C. chinense, Artemisia annua, C. pseudophragmites, S. brachyotus, Artemisia subdigitata, A. sacrorum, I. chinense, A. fatua, S. collina* | 86% |
| TA-OR | *P. tabulaeformis, I. sonchifolium, A. hedinii, C. chinense, S. viridis, S. brachyotus, Toraxacum mongolicum, A. sacrorum, I. chinense, S. collina* | 90% |
| MF-CK | *S. matsudana*, *S. Chinensis, I. sonchifolium, A. pendula, C. chinense, A. sacrorum, S. collina* | 90% |
| MF-IN | *S. matsudana*, *S. Chinensis, I. sonchifolium, Plantago asiatica, A. pendula, Calystegia hederacea, C. chinense, P. communis, A. sacrorum, S. collina* | 95% |
| MF-IO | *S. matsudana*, *S. Chinensis, I. sonchifolium, P. asiatica, A. pendula, S. brachyotus, A. sacrorum, C. setosum, S. collina* | 95% |
| MF-OR | *S. matsudana*, *S. Chinensis, I. sonchifolium, C. hederacea, C. chinense, S. japonica, H. hispidus, L. bicolor, A. sacrorum, I. chinense, S. collina* | 95% |
