# Supplementary material for: Effects of Different Regeneration Scenarios and Fertilizer Treatments on Soil Microbial Ecology in Reclaimed Opencast Mining Areas on the Loess Plateau, China
Source: PLoS One. 2013 May 2;8(5):e63275. doi: 10.1371/journal.pone.0063275 (PMC3642173; doi:10.1371/journal.pone.0063275)
Supplement: Table S2 — Results of three-way ANOVA showing the effects of season, regeneration scenarios and fertilizer treatments for Soil bacteria, archaea, fungi and total microbe RFs richness index ( S ), Shanon-Wiener diversity index ( H’ ), Simpson diversity index ( D ) and Pielou evenness index ( E ). ** Effect is significant at the 0.01 level; * Effect is significant at the 0.05 level. ns Effect is not significant. (DOC) [file pone.0063275.s005.doc]

|  | **Bacteria** | | | | **Archaea** | | | | **Fungi** | | | | **Total microbe** | | | |
| --- | --- | --- | --- | --- | --- | --- | --- | --- | --- | --- | --- | --- | --- | --- | --- | --- |
|  | ***S*** | ***H’*** | ***D*** | ***E*** | ***S*** | ***H’*** | ***D*** | ***E*** | ***S*** | ***H’*** | ***D*** | ***E*** | ***S*** | ***H’*** | ***D*** | ***E*** |
|  | *Hae*III | | | | | | | | | | | | | | | |
| Season | ns | ns | ns | ns | ** | ** | ** | ** | ** | ** | ** | ** | ** | ** | ** | ** |
| Scenarios | ns | ns | ns | ns | ns | ** | ** | ** | ** | ** | * | ** | ns | ** | * | ** |
| Fertilizer | ns | ns | ns | ns | ns | * | ** | ** | ns | ns | ns | ns | ns | ns | ns | ns |
| Season × Scenarios | ns | ns | ns | ns | ns | ** | ** | * | ** | * | ns | * | ns | ns | ns | ns |
| Season × Fertilizer | * | ns | * | ns | ns | ns | ns | ns | ns | ns | ns | ns | ns | * | ns | * |
| Scenarios × Fertilizer | ns | ns | ns | ns | ns | ** | ** | ** | ns | * | ** | ** | ns | ns | ns | ns |
| Season × Scenarios × Fertilizer | ns | ns | ns | ns | ns | * | * | * | ns | ns | ns | ** | ns | ns | ns | ns |
|  | *Msp*I | | | | | | | | | | | | | | | |
| Season | ** | ** | ** | * | ** | ** | ** | ** | ** | ** | ** | ** | ** | ** | ** | ** |
| Scenarios | * | ns | ns | ** | ns | ns | * | * | ns | ns | ns | * | ns | * | ns | ** |
| Fertilizer | ns | ns | ** | ns | ns | ns | ** | ns | ns | ns | ns | ns | ns | ns | * | ns |
| Season × Scenarios | * | ns | ns | ns | * | * | * | ** | ** | * | ns | ns | * | ** | ns | * |
| Season × Fertilizer | ** | ** | ** | * | ns | ns | ns | ns | ns | ns | ns | ns | ** | ** | ** | * |
| Scenarios × Fertilizer | ns | ns | ns | * | * | ** | ** | * | ns | ns | ns | ns | ns | ns | * | ** |
| Season × Scenarios × Fertilizer | ns | ns | ns | * | ns | * | ** | * | * | ns | ns | ns | ns | ns | ns | ns |
