# Supplementary material for: Effects of Different Regeneration Scenarios and Fertilizer Treatments on Soil Microbial Ecology in Reclaimed Opencast Mining Areas on the Loess Plateau, China
Source: PLoS One. 2013 May 2;8(5):e63275. doi: 10.1371/journal.pone.0063275 (PMC3642173; doi:10.1371/journal.pone.0063275)
Supplement: Table S3 — Ratios of positive and negative association from Spearman rank correlation test of the inter-species correlation of plant and inter-RFs correlation of soil bacteria, archaea, fungi and total microbes in the reclaimed mining area. CO, SA, TA and MF represent Lotus corniculatus, Medicago sativa, Pinus tabulaeformis and Salix matsudana–Sabina chinensis mixed forest. CK, IN, IO and OR represent no, inorganic, organic and a combination of inorganic and organic fertilizer added to soils. (DOC) [file pone.0063275.s006.doc]

|  | **Plant** | **Bacteria** | **Archaea** | **Fungi** | **Total microbes** |
| --- | --- | --- | --- | --- | --- |
| CO-CK | 0.63 | 0.70 | 0.50 | 0.64 | 0.90 |
| CO-IN | 0.73 | 0.73 | 0.64 | 0.71 | 0.93 |
| CO-IO | 0.78 | 0.81 | 0.73 | 0.71 | 0.99 |
| CO-OR | 0.64 | 0.78 | 0.57 | 0.76 | 1.04 |
| SA-CK | 0.25 | 0.80 | 0.33 | 0.78 | 0.84 |
| SA-IN | 0.50 | 0.82 | 0.73 | 0.80 | 0.70 |
| SA-IO | 0.25 | 0.94 | 0.57 | 0.82 | 0.98 |
| SA-OR | - | 0.84 | 0.89 | 0.91 | 0.94 |
| TA-CK | 0.50 | 0.71 | 0.50 | 0.71 | 0.91 |
| TA-IN | 0.68 | 0.82 | 0.50 | 0.77 | 0.95 |
| TA-IO | 0.62 | 0.71 | 0.87 | 0.89 | 0.96 |
| TA-OR | 0.61 | 0.87 | 0.50 | 0.76 | 0.92 |
| MF-CK | 0.40 | 0.74 | 0.65 | 0.71 | 0.88 |
| MF-IN | 0.44 | 0.70 | 0.71 | 0.94 | 0.97 |
| MF-IO | 0.50 | 0.74 | 0.54 | 0.76 | 1.18 |
| MF-OR | 0.45 | 0.93 | 0.74 | 0.81 | 0.93 |
